# Schoolgirls' Food and Work

**Published:** 1893-02-11

**Authors:** 


### The Hospital

An Institutional Journal of

Science, Medicine, Nursing, and Philanthropy.

*For Hospitals, Asylums, Infirmaries, Dispensaries, Medical Practitioners, Students, Nurses, and the Charitable Public.*

Vol. XIII.—No. 333.] [Feb. 11, 1893.

### Schoolgirls' Food and Work.

An article published in The Hospital on the subject of the feeding of schoolgirls has excited a good deal of comment in various quarters. The *Evening Standard* considers that probably schoolgirls alone will "feel unalloyed gratitude" to us; and the *Birmingham Post* pathetically asks us to suggest the best methods of furnishing the wherewithal to carry out our counsels of perfection. Newspaper writers, parents, and schoolmistresses will perhaps bear with us if we enter upon a little fuller and more fundamental consideration of that part of the subject where the shoe seems most sharply to pinch.

In the first place, the facts of physiology are what they are by virtue of Nature's fiat: they are not creations of the doctor. The business of the physiologists is to observe, to understand, and to expound. He can only observe, understand, and expound what he actually finds. If he were to say what people wish rather than what the facts of physiology command, he would be found a false witness of science, an untrustworthy and wicked man. This is clear as noonday. Take an illustration. "Thou shalt not steal," says the eighth commandment. If a clergyman preach from this text, even to a congregation of robbers, he must preach the full and unimpaired truth. If he were to begin to whittle it away, and to say "Thou shalt not steal often," or "Thou shalt not steal much," or "Thou shalt not steal except under great temptation," his congregation of robbers would be the first to laugh at him. It is precisely the same with physiology. The physician, who is the priest of the physiological cult, is under the most absolute compulsion to preach exactly the facts and doctrines he finds, and nothing else. Doubtless they are often found to be "counsels of perfection," even by the initiated. But notwithstanding that, he must preach them with unbending rectitude, and the great world must learn to obey his precepts as quickly and as fully as it can, or take the consequences.

Our demands on behalf of schoolgirls amount to this and nothing more; that they shall be placed in the best possible circumstances for developing the fullest possible measure of health and happiness of which they are capable. But is this a scientific gospel of good news? Undoubtedly it is! If science has a gospel, why should it not be preached? And if it be a worthy gospel, why should not its preachers preach it with enthusiasm; aye, and with insistance too? Let us see now if this gospel be practicable! One of physiology's demands is that schoolgirls shall be provided with a "liberal supply of meat twice a day." Well! what constitutes a liberal supply for a schoolgirl of fifteen? Half-a-pound a day, or a quarter of a pound for each of the two meat meals, would be ample—more, indeed, than enough. But what is the cost of half-a-pound of good English or Scotch beef when the second best as well as the best cuts are bought, and the meat is purchased in wholesale quantities as the schoolmistress buys it? It cannot be more than fivepence at the outside; and it ought not to be so much. Fivepence a day, then, is clearly more than enough. Now add to this one fresh egg a day, which, bought at wholesale prices, will not cost more than a penny all the year round; bread a penny, a pint of new milk twopence, vegetables a penny, and fruit twopence. Here is a daily dietary which, in our judgment, is amply sufficient, and more than sufficient for the average schoolgirl. If tea and coffee be required, they can take the place of a portion of the pint of milk; and if fish be provided, it can be employed as a substitute for meat or eggs. The twopence a day allowed for fresh fruit will provide jam and butter by way of change from the fresh fruit. In short, for one shilling a day everything can be provided on the most liberal scale for the satisfaction of every physiological necessity. Now, there are only seven days in the week, and forty weeks in the school year; therefore, the total cost of feeding a schoolgirl with half-a-pound of meat a day, and everything else to match, amounts to the modest sum of fourteen pounds a year.

But is it not the case that the school fees for the class of girls now under discussion range from seventy pounds a year upwards? Is it not also the case that governesses in girls' schools receive very small salaries; that extra fees are charged for all manner of extra subjects, and that a generous profit is realized on innumerable school books? The truth is that a girls' school, when the business is conducted by a business-like woman, is exceedingly profitable. The particular schoolmistress who recently had charge of the writer's own little girl fed her pupils even more liberally than is here demanded; and not long ago was able to erect at her own sole cost a handsome memorial window to a deceased vicar of the parish.

Let us examine the case of innumerable schools whose charges range, not from seventy pounds a year upwards, but from thirty pounds a year? Is physiology so stern a dictatrix as to demand from these exactly the same obedience as from the others? The answer to this is that physiology demands for her rewards of perfect health exactly the same obedience to law from all sorts and conditions of men, women, and children. This is a rule which cannot be too often and too sternly insisted upon. But there are more ways than one of obeying physiological laws, just as there are of obeying other laws. We do not hesitate to say that a thoroughly clever and managing woman can supply all the meat, eggs, fish, vegetables, and fruit that are indispensably necessary, for two-thirds of the cost already indicated, that is for less than ten pounds a year. But in order to do this there must be a buyer in the market and a cook in the kitchen. School keeping demands not only the power to teach, but the business faculty to buy food, and a practical knowledge of scientific cookery. If it be said that to write like this is unpractical, the answer is, that in that case physiology herself must be called unpractical. Physiology never asks where the clever women are to be found, who are to manage schools so excellently, nor how the food is to be supplied which is to give the children health. She silently but absolutely refuses to paint the bloom of health on any cheek whatever without an abundant supply of suitable food, pure air, healthy exercise, sound sleep, and all the rest of it. We may think physiology very cruel for all this, but she does not mind our criticisms in the least. To the physician's eye the law is writ as clear and plain as if it were graved with steel across the midnight sky: "Do this, and be well; do that, and be ill and miserable, and die before your time."

It has been complained that our suggestions for study, which permitted six hours a day as the maximum, even for strong girls, will not work in practice. Our contention is that four hours of real study a day, with an hour in the evening for preparation, and preparation assisted by competent evening tutors, will produce better results over a course of years than twice the amount of time spent in dull routine. The writer is not only a physician, he was at one time, and that for a considerable period, a tutor and a "coach." He knows by experience what he is talking about. He insists that even from the point of view of study, four hours a day in school, with an hour for evening preparation, will produce better school results than six hours a day in school, and two hours for evening preparation, over a course of years.

But besides, what is it we want to make of our girls; that is, of our ordinary girls? We want to make them practical, thoughtful, healthy, happy, and domesticated women. Perhaps one in ten of them will have to earn her living as a governess. But are we to sacrifice the nine to the one? The girls who are to be governesses must be taught separately, and even they must be fitted for their task, not by extra hours in school, but by additional years. The whole subject is urgently important, and there seems to be an amount of unphysiological and dangerous mismanagement prevalent in girls' schools which makes the ardent physiologist positively stand aghast. The average schoolmistress is to the physiologist what the average African is to the missionary—an uninstructed dweller in the "dark places of the earth." Surely parents, and even rightminded schoolmistresses themselves, will "lend a hand," as the sailors say, to help physiologists to mend all this.

